# Underwater endoscopic mucosal resection of a follicular lymphoma: A case report

**DOI:** 10.1097/MD.0000000000027610

**Published:** 2021-10-29

**Authors:** Tae Un Kim, Su Jin Kim, Cheol Woong Choi

**Affiliations:** a Department of Radiology Yangsan, Korea; b Department of Internal Medicine, Pusan National University School of Medicine and Research Institute for Convergence of Biomedical Science and Technology, Pusan National University Yangsan Hospital, Yangsan, Korea.

**Keywords:** Endoscopic mucosal resection, Gastric neoplasm, Lymphoma

## Abstract

**Introduction:**

Endoscopic resection of a follicular lymphoma (FL) presenting as a gastric subepithelial tumor (SET), along with periodic follow up can be a treatment option because gastrointestinal FL cells tend to reside in the primary site, which may explain its indolent nature.

**Patient concerns:**

A gastric lesion was found incidentally during a screening endoscopy in 73-year-old woman without any gastrointestinal symptom.

**Diagnosis:**

The patient was diagnosed with a grade I FL that was 1.4 cm large, at the greater curvature of lower-body.

**Intervention:**

We performed underwater endoscopic mucosal resection (UW-EMR), and there was no serious complication, such as bleeding and perforation.

**Outcomes:**

Complete en bloc resection was achieved with UW-EMR. Follow-up endoscopic biopsy after 3 months revealed no residual tumor on the resection site.

**Conclusion:**

UW-EMR may be a simple and safe resection method for gastric FL without metastases, that measure >1 cm.

## 1 Introduction

Follicular lymphoma (FL) is the most common form of non-Hodgkin lymphoma. In FL, tumor cells originate from germinal center B cells; therefore, FL predominantly occurs at lymph nodes. Primary gastrointestinal FL is extremely rare, among the several cases of primary extranodal FL.^[1]^ Most cases of gastrointestinal FL occur in the small intestine, especially in the duodenum, followed by the jejunum. Since primary gastrointestinal FLs mostly appear as subepithelial tumors (SETs), endoscopic forceps biopsies are often inconclusive.^[2]^

Endoscopic submucosal dissection (ESD) for gastric SETs measuring more than 1 cm requires prolonged operative time and an experienced therapeutic endoscopist.^[3]^ UW-EMR has been described as an effective and safe treatment modality for SETs embedded in the submucosa.^[4–6]^ Water immersion during underwater endoscopic mucosal resection (UW-EMR) decreases the luminal extension force, increases the mucosal and submucosal buoyancy, and keeps the muscularis propria circular behind the submucosa.^[7]^ A water-filled stomach makes tumors to float without submucosal injection and facilitates snaring of the tumor by the creation of a pseudopedicle.^[8]^

## 2 Case report

**Figure 1:**
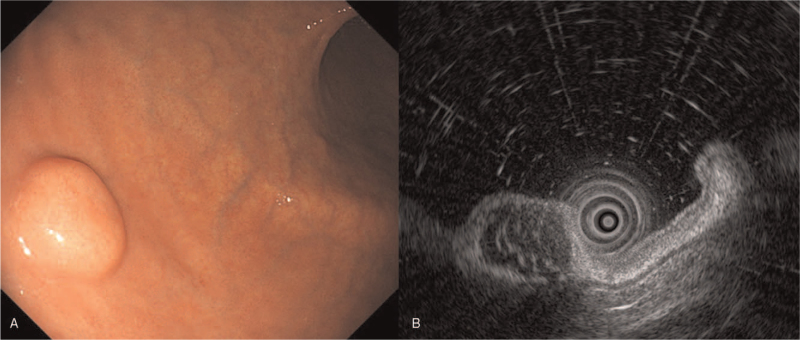
(A) A wide-based and sessile subepithelial tumor covered with normal gastric mucosa is found incidentally on the greater curvature of the stomach body during screening endoscopy. (B) Endoscopic ultrasonography reveals a heterogeneous hypoechoic lesion of 1.4 X 0.5 cm in the submucosal layer.

**Figure 2:**
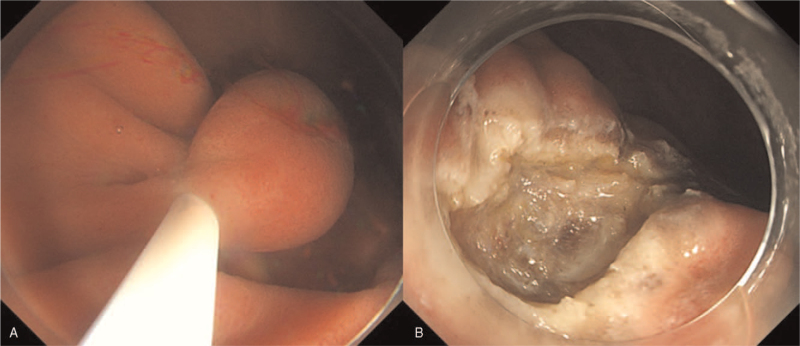
(A) Water immersion makes the tumor to float from the proper muscle and capture the base of lesion easily by 25 mm polypectomy snare. (B) There is no evidence of residual tumor around the resected port of the lesion.

**Figure 3:**
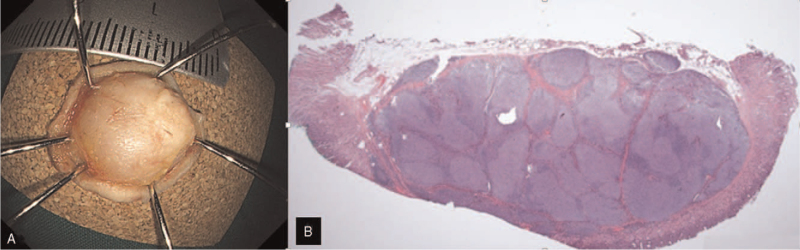
(A) En-bloc and grossly R0 resection are achieved. (B) Histologic findings are nodular aggregates of small lymphoid cells in the submucosa with little mucosal involvement (H&E ×20).

A 73-year-old woman was referred to our hospital to undergo an endoscopic ultrasonography (EUS) for a gastric SET that was covered with normal gastric mucosa, which was located on the greater curvature, at the lower part of the gastric body; it was found incidentally during screening endoscopy (Fig. 1A). EUS showed a heterogeneous hypoechoic lesion measuring 1.4 × 0.5 cm in the submucosal layer (Fig. 1B). Endoscopic forceps biopsies were inconclusive. An abdominal computed tomography showed a soft tissue density subepithelial tumor without lymph node enlargement. We decided to perform an endoscopic resection for diagnosis and treatment. ESD requires prolonged operative time and an experienced therapeutic endoscopist. Recently, UW-EMR has been described as an effective and safe treatment modality for SETs embedded in the submucosa. Therefore, UW-EMR (video) was performed using a 25-mm polypectomy snare (Endo-Flex GmbH, Germany) through a single channel endoscope (Olympus GIF-HQ290, Tokyo, Japan) using ENDO CUT Q current (effect 3, cut duration 2, cut interval 3), which was generated using the VIO 300 D electrosurgical unit (Erbe Elektromedizin GmbH, Tübingen, Germany) (Fig. 2A). En bloc resection was achieved and the operative time was 5 minutes. There were no signs of bowel perforation or residual lesions (Fig. 2B), and the resection margin was grossly tumor-free (Fig. 3A). The histological diagnosis was a grade I FL which measured 1.4 cm (Fig. 3B) with tumor-free margins. Follow-up endoscopic biopsy after 3 months revealed no residual tumor on the resection site. The patient was planned to undergo periodic surveillance using endoscopy and abdominal computed tomography without additional treatment. The patient provided informed consent for the publication of her clinical data accompanying images.

## 3 Discussion

FL involving the gastrointestinal tract by may be rare. Most reported cases of primary gastric FL presented with a localized disease and single or multiple submucosal tumors.^[2]^ Gastric FL presenting as SET is uncommon and its incidence rate has not been reported.^[1]^ A primary gastric FL may be misdiagnosed as an extra-nodal marginal zone B-cell lymphoma on biopsy specimens.^[2]^ The overlying normal mucosa over the FL presenting as a SET could hinder the accurate diagnosis through endoscopic mucosal biopsy. Most small SETs covered with normal mucosa are identified during periodic endoscopic follow-up. In SETs that are >1 cm in size, EUS can help physicians to decide the management strategy through details such as the origin of the layers and the echogenicity of the tumor.^[9]^ The EUS findings in this case revealed a hypoechoic lesion that originated from the submucosal layer, and biopsy findings were inconclusive. Hypoechoic SETs that originate from the submucosal layer include neuroendocrine tumors (NETs), lymphomas, and adenocarcinomas. Therefore, tissue diagnosis of submucosal layer origin SETs with hypoechogenicity is important to decide the treatment strategy.

Conventional endoscopic mucosal resection (EMR) is a simple method that can be used to diagnose and treat small SETs embed in the submucosal layer. Submucosal injections during the procedure sometimes makes EMR difficult due to the cushion effect of the injected fluid.^[10]^ ESD is a good alternative that can be used to overcome the limitations of conventional EMR.^[11,12]^ The disadvantages of ESD are prolonged procedure time, technical burden, and a higher incidence of adverse event, such as perforation compared to EMR. Systemic review and meta-analysis of UW-EMR versus conventional EMR for the treatment of colorectal polyps revealed that UW-EMR was associated with an increase in en-bloc resection rate (odds ratio 1.84; 95% confidence interval 1.42–2.39).^[13]^ A recent study reported that UW-EMR was effective in removing rectal NETs and showed similar treatment outcomes and a short procedure time compared to ESD.^[4]^ There is also a report of successful UW-EMR for an esophageal granular cell tumor that was >1 cm.^[14]^ Therefore, we performed UW-EMR for a gastric SET that was >1 cm in diameter and achieved complete resection. The patient's final diagnosis was a grade I FL measuring 1.4 cm.

This is the first reported case of the removal of a gastric FL using the UW-EMR technique. We proposed that gastric lesions that originate from the mucosa or submucosa and are <1.5 cm in size can be an indication for UW-EMR. SET originated proper muscle is contraindication of EMR. Small gastrointestinal SETs sometimes can be hard to capture during UW-EMR. However, the complication rate was not significantly different between UW-EMR and conventional EMR.^[13]^

Heterogeneous treatment modalities, such as radiotherapy, chemotherapy, and surgery are used in the treatment of FL. In contrast to nodal FL, tumor cells in gastrointestinal FL tend to reside in the primary site and have an indolent nature. Therefore, the “watch and wait” strategy can be an option after the resection of the primary gastrointestinal FL. A previous report on performing endoscopic resections for gastric FL without lymph node enlargement on abdominal computed tomography showed that there was no recurrence after complete resection.^[15]^

In conclusion, UW-EMR appears to be a simple and safe resection method for gastrointestinal FL without metastases that measure more than 1 cm.

## Acknowledgments

This work was supported by 'Supporting Project to evaluation New Domestic Medical Devices in Hospitals’ funded by ’Ministry of Health and Welfare (MOHW)’ and ’Korea Health Industry Development Institute (KHIDI)’.

## Author contributions

**Conceptualization:** Su Jin Kim.

**Data curation:** Tae Un Kim, Cheol Woong Choi.

**Supervision:** Cheol Woong Choi.

**Writing – original draft:** Tae Un Kim.

**Writing – review & editing:** Su Jin Kim.

## Supplementary Material

Supplemental Digital Content

## References

[1] Herrmann R Panahon AM Barcos MP. Gastrointestinal involvement in non-Hodgkin's lymphoma. Cancer 1980;46:215–22. 7388763 10.1002/1097-0142(19800701)46:1<215::aid-cncr2820460136>3.0.co;2-6

[2] Na HY Kim YA Lee C. Gastric follicular lymphoma: a report of 3 cases and a review of the literature. Oncol Lett 2018;16:741–8. 29963140 10.3892/ol.2018.8744 PMC6019973

[3] Nonaka S Oda I Tada K. Clinical outcome of endoscopic resection for nonampullary duodenal tumors. Endoscopy 2015;47:129–35. 25314330 10.1055/s-0034-1390774

[4] Park SS Han KS Kim B. Comparison of underwater endoscopic mucosal resection and endoscopic submucosal dissection of rectal neuroendocrine tumors (with videos). Gastrointest Endosc 2020;91: doi: 10.1016/j.gie.2019.12.039]. 10.1016/j.gie.2019.12.039 31904380

[5] Rezende DT Kawaguti FS Safatle-Ribeiro AV. Underwater endoscopic resection of an ileal neuroendocrine tumor. Endoscopy 2021;53:E48–9. 32503073 10.1055/a-1178-0143

[6] Anderloni A Murino A Jovani M. Underwater endoscopic mucosal resection of a duodenal neuroendocrine tumor. Gastrointest Endosc 2016;83:259–60. 26264433 10.1016/j.gie.2015.08.001

[7] Yamashina T Uedo N Akasaka T. Comparison of underwater vs conventional endoscopic mucosal resection of intermediate-size colorectal polyps. Gastroenterology 2019;157: 451-461e452. 10.1053/j.gastro.2019.04.005 30981791

[8] Iwagami H Kanesaka T Ishihara R. Underwater endoscopic mucosal resection for remaining early gastric cancer after endoscopic submucosal dissection. Endoscopy 2019;51:E229–30. 30995684 10.1055/a-0875-3429

[9] Hwang JH Rulyak SD Kimmey MB. American Gastroenterological Association Institute technical review on the management of gastric subepithelial masses. Gastroenterology 2006;130:2217–28. 16762644 10.1053/j.gastro.2006.04.033

[10] Binmoeller KF Shah JN Bhat YM. Underwater” EMR of sporadic laterally spreading nonampullary duodenal adenomas (with video). Gastrointest Endosc 2013;78:496–502. 23642790 10.1016/j.gie.2013.03.1330

[11] Lee DS Jeon SW Park SY. The feasibility of endoscopic submucosal dissection for rectal carcinoid tumors: comparison with endoscopic mucosal resection. Endoscopy 2010;42:647–51. 20669076 10.1055/s-0030-1255591

[12] Sato Y Takeuchi M Hashimoto S. Usefulness of endoscopic submucosal dissection for type I gastric carcinoid tumors compared with endoscopic mucosal resection. Hepatogastroenterology 2013;60:1524–9. 23933946 10.5754/hge121185

[13] Choi AY Moosvi Z Shah S. Underwater versus conventional EMR for colorectal polyps: systematic review and meta-analysis. Gastrointest Endosc 2021;93:378–89. 33068608 10.1016/j.gie.2020.10.009

[14] Hwang CS Kim SJ Choi CW. Underwater endoscopic mucosal resection for esophageal granular cell tumor sized more than 1 cm. Dig Endosc 2020;32:995. 32512623 10.1111/den.13764

[15] Kim HJ Choi CW Park SB. Gastric follicular lymphomas presenting as subepithelial tumors: two cases. Korean J Helicobacter Up Gastrointest Res 2018;18:06.

